# Modulation of T cell function and survival by the tumor microenvironment

**DOI:** 10.3389/fcell.2023.1191774

**Published:** 2023-05-18

**Authors:** Nikita Mani, Dathan Andrews, Rebecca C. Obeng

**Affiliations:** ^1^ Department of Pathology, Northwestern University Feinberg School of Medicine, Chicago, IL, United States; ^2^ Department of Pathology, Case Western Reserve University School of Medicine, Cleveland, OH, United States; ^3^ Case Comprehensive Cancer Center, Case Western Reserve University School of Medicine, Cleveland, OH, United States; ^4^ University Hospitals Cleveland Medical Center, Cleveland, OH, United States

**Keywords:** T cell function, immunosuppression, metabolism, tumor microenvironment, immune checkpoint

## Abstract

Cancer immunotherapy is shifting paradigms in cancer care. T cells are an indispensable component of an effective antitumor immunity and durable clinical responses. However, the complexity of the tumor microenvironment (TME), which consists of a wide range of cells that exert positive and negative effects on T cell function and survival, makes achieving robust and durable T cell responses difficult. Additionally, tumor biology, structural and architectural features, intratumoral nutrients and soluble factors, and metabolism impact the quality of the T cell response. We discuss the factors and interactions that modulate T cell function and survive in the TME that affect the overall quality of the antitumor immune response.

## Introduction

It is well-established that the immune system plays a major role in tumor control. Presentation of tumor-associated antigens (or lack thereof) can activate the immune system to recognize and eliminate dysfunctional and aberrant tumor cells. From decades of research, we appreciate the complexity of the immune machinery, which involves all aspects of the immune system from innate to adaptive responses, that acts as a defense mechanism against tumor growth. Dedicated and persistent efforts by many investigators have ushered in a new era for the clinical management of cancer and has brought the immune system to the forefront of clinical medicine for patients with cancer. Therapies targeting all components of the immune system, also known as immunotherapy and once significantly underappreciated, are now the standard of care for many patients with different types of tumors. The preponderance of evidence supports the CD8 T cell as the major subset of immune cells that is critically important for antitumor immunity and clinical efficacy in preventing disease progression and extending overall survival.

**FIGURE 1:**
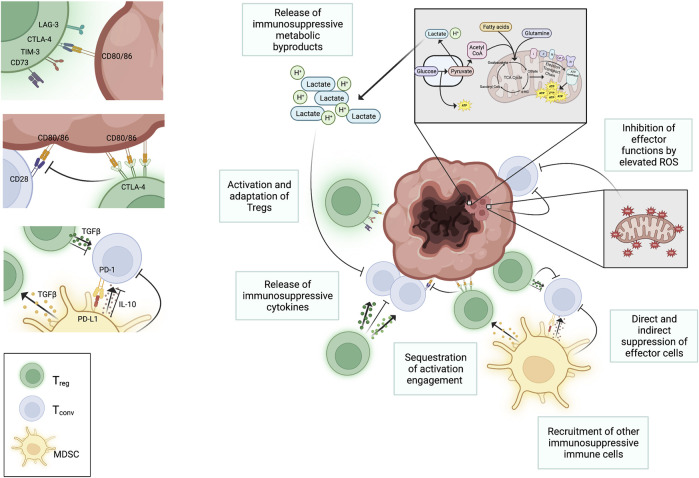
Negative regulation of T cell function in the TME. Several factors in the TME negatively impact T cell function. High metabolic activity of tumors metabolism and increased mitochondrial activity results in accumulation of harmful metabolic byproducts in the interstitial space that negatively impacts T cells in the TME. Regulatory immune cells, like T regs and myeloid derived suppressor cells (MDSCs), are recruited to the sites of tumors and suppress proliferation and function of conventional T cells through both the engagement of checkpoint inhibitors (e.g., PD-1 on conventional T cells) and through the release of suppressive cytokines (e.g., TGFβ, IL-10, and IL-35). These cytokines also serve to further activate T regs. Activated T regs upregulate CTLA-4, which selectively binds to CD80 and CD86 on APCs blocking interaction between CD80/86 and CD28 on conventional T cells. This hinders T cell activation. The binding of CTLA-4 to CD80 and CD86 can also initiate the endocytosis of these ligands further decreasing availability for binding to CD28 on conventional T cells. Despite the buildup of metabolic waste that suppress conventional T cells in the TME, regulatory immune cells are better able to tolerate the harsh conditions and can continue to extend their suppressive roles against the anti-tumor effector functions of conventional T cells. Created with Biorender.com.

While the lymphoid compartments generally act as the sites for the initiation and development of antitumor immunity, the TME exerts significant influence on the quality of the immune response (Figure 1). In addition to the TME recruiting immune cells and supporting *de novo* T cell activation, it is generally an immunosuppressive environment. This suppressive environment poses challenges and limits the clinical efficacy of immunotherapy. In this review, we will discuss how T cells function and survive in the TME with an emphasis on CD8 T cells. We will also address challenges in maintaining an effective T cell response and opportunities to improve the clinical efficacy of antitumor immunity.

## T cell activation and development of effector function

Antigens derived from tumor cells may be first encountered in the interstitial fluid that drains from lymphatic vessels and into lymph nodes. Proteins and peptides are taken up by immune scavengers and specialized antigen presenting cells (APCs) such as dendritic cells (DCs). These APCs process and present the proteins and peptides derived from the tumor cells and the TME on major histocompatibility complexes (MHC) I and II by direct priming or through a process known as cross-presentation (Huang et al., 1994; Wolkers et al., 2001; Hargadon et al., 2006; McDonnell et al., 2010). Although macrophages (Pfeifer et al., 1993; Rock et al., 1993), neutrophils (Tvinnereim et al., 2004), B cells (Ke and Kapp, 1996), and DCs (Chung et al., 2005; McDonnell et al., 2010) can cross-present, the process is most efficiently carried out by DCs expressing CD8a (den Haan et al., 2000; Belz et al., 2004; Allan et al., 2006) and CD103 (Bedoui et al., 2009; del Rio et al., 2007). Recognition of the MHC-peptide complexes by naïve T cells and engagement of costimulatory receptors by ligands on the activated APCs results in a signaling cascade that leads to T cell activation, clonal expansion supported by interleukin 2 (IL-2), and the differentiation into effector T cells (Smith-Garvin et al., 2009). Cancer cells that have metastasized into lymph nodes can also directly present antigens to T cells, but this interaction is generally suboptimal in activating naïve T cells due to the lack of co-stimulatory ligand expression by the cancer cells. The TME can also serve as a site for the initial activation of naïve T cells. In experiments using mice that lack lymph nodes and adoptive transfer of naïve T cells, investigators have shown that the TME can support *de novo* naïve T cell activation and differentiation into effector T cells (Yu et al., 2004; Thompson et al., 2010).

T cell activation is negatively regulated by inhibitory molecules, the most extensively studied being cytotoxic T lymphocyte-associated antigen 4 (CTLA-4). It is constitutively expressed on T cells and binds to the same ligands (CD80 and CD86) as CD28 (Linsley et al., 1991; Azuma et al., 1993; Freeman et al., 1993; Hathcock et al., 1993; Linsley et al., 1994). The affinity of CTLA-4 for CD80 and CD86 is significantly higher than that of CD28 and the general dogma is that CTLA-4 competes with CD28 to modulate the amplitude of TCR signaling during CD4 and CD8 T cell activation (Linsley et al., 1994; Egen and Allison, 2002; Riley et al., 2002; Schneider et al., 2002; Parry et al., 2005; Schneider et al., 2006). In binding to its ligands, CTLA-4 sequesters CD80 and CD86 and may actively remove the ligands from the surface of APCs further limiting CD28 engagement with the ligands (Qureshi et al., 2011). Aside from its inhibitory effects, CTLA-4 is a downstream transcriptional target of FOXP3 and its expression on regulatory T cells (T regs) enhances the suppressive activity of this subset of T cells (Fontenot et al., 2003; Hori et al., 2003; Wing et al., 2008; Peggs et al., 2009).

## Tumor antigens

Recognition of antigens presented by tumor cells is critical for an effective T cell response. Tumors arise from normal cells in which genetic alterations lead to uncontrolled proliferation and transformation into malignant cells (Hanahan and Weinberg, 2000). As such antigens presented by non-viral tumors are of self-proteins or variations thereof. Identification of tumor antigens can be accomplished in several ways. They can be identified using serological analysis of recombinant tumor cDNA expression library (Kawakami et al., 1994) or synthetic peptide libraries (Townsend et al., 1986) by T cell clones. Alternatively, peptides can be isolated from MHC molecules and analyzed by mass spectrometry (Hunt et al., 1992). Over 170 antigenic peptides derived from 60 human antigens that are recognized by T cells have been identified (Renkvist et al., 2001). These antigens are generally classified into four groups: 1) cancer/testis or germline antigens, 2) tissue-specific differentiation proteins, 3) mutated self-proteins, and 4) those derived from post-translational modifications (PTMs) of proteins such as phosphopeptides (Boon and van der Bruggen, 1996; Zarling et al., 2000; Zarling et al., 2006; Mohammed et al., 2008; Depontieu et al., 2009; Zarling et al., 2014). Despite the fact that many tumor antigens are of unmutated self-proteins, CD8 (van der Bruggen et al., 1991; Cox et al., 1994; Van den Eynde et al., 1995) and CD4 (Topalian et al., 1994; Chaux et al., 1999; Pieper et al., 1999) T cell responses against antigens in all classes can be generated, sometimes spontaneously in humans (Bakker et al., 1994; Kawakami et al., 1994; Robbins et al., 1996).

The amount of tumor antigen present can modulate immune surveillance. Suboptimal activation of T cells may occur due to the lack of the necessary costimulatory molecules on tumor cells or because of induction of APCs that are ineffective at stimulating T cells (Gabrilovich et al., 1996; Menetrier-Caux et al., 1998; Munn et al., 2004; Hargadon et al., 2006). In situations where antigen is limited, highly avid CD8 T cells, which require only small amounts of antigen for activation, are needed to generate productive immune responses. Otherwise, those tumors go unnoticed by the immune system (Dudley et al., 1999; Zeh et al., 1999). Several studies in mice (Urban et al., 1986; Shankaran et al., 2001) and in humans (Slingluff et al., 2000; Yamshchikov et al., 2005) have shown that an ongoing immune response can place selective pressure that leads to the emergence of antigenic loss variants of tumors that escape immune recognition. This suggests that some tumor antigens are dispensable and not functionally important for tumor formation and/or metastasis. It also illuminates the need to identify and target functionally relevant tumor antigens for cancer therapy (Hirohashi et al., 2009). Antigens derived from proteins that regulate the malignant properties of tumors are necessary because such antigens are needed to maintain the malignant phenotype of tumors. Additionally targeting such antigens may counteract the emergence of antigenic loss variants of tumors as downregulation of the proteins would not be beneficial to the tumor. Systematic evaluation and careful selection of tumor antigens that are 1) differentially expressed in tumors and minimally expressed in normal cells, 2) functionally relevant to malignancy, and 3) antigenically distinct and immunogenic should provide a collection of peptides that will be more efficacious in clinical trials.

## T cell differentiation and function

In the process of differentiation, T cells acquire certain properties and attributes that dictate their functional capacity. In the context of effector function, CD4 T cells can differentiate into several different subsets (Murphy and Stockinger, 2010; Zhu, 2018). T_H_1 that produce interferon gamma (IFNγ) and tumor necrosis factor (TNF) to activate macrophages and recruit leukocytes. T_H_2 subsets that secrete IL-4 and IL-13 and stimulate mast cells and eosinophils. Other subsets such as T regs hinder effective T cell response (Kim and Cantor, 2014). The differentiation of CD4 T cells into various subsets is heavily influenced by the cytokines present in the microenvironment. Polarization of CD4 T cells into the proinflammatory T_H_1 phenotype is mediated by IL-12 and IFNγ (Hsieh et al., 1993; Perez et al., 1995) while T_H_2 polarization is regulated by IL-2 and IL-4 (Le Gros et al., 1990; Swain et al., 1990). T_H_17 CD4 T cell differentiation is mediated by transforming growth factor beta (TGFβ), IL-6, IL-21 and/or IL-23 (Veldhoen et al., 2006; Zhou et al., 2007). T regs comprise natural and inducible T regs cells that require TGFβ and IL-2 for differentiation (Chen et al., 2003; Davidson et al., 2007; Takeuchi and Nishikawa, 2016). Both natural and inducible T regs can suppress effector function of CD4 and CD8 T cells through direct cellular interactions and through the release of cytokines and growth factors such as IL-10, IL-35, and TGFβ that negatively regulate effector function. For example, IL-10 has been shown to suppress T_H_17 effector cytokine production, while TGFβ dampens the effector response by regulating T_H_1 and T_H_17 differentiation and subsequent T cell tolerance (Li et al., 2007; Chaudhry et al., 2011). These cytokines have also been shown to block antigen presentation and interactions between conventional T cells and APCs in the TME. CD8 T cells differentiate into cytotoxic cells with the ability to directly kill tumor cell targets by producing cytokines and producing lytic granules including granzymes and perforin (Breart et al., 2008; Schietinger et al., 2013). Some subsets of T cells differentiate into circulatory effectors that move through tissues, lymphatics, and blood while others acquire features that lead to their retention within tissues. The acquisition of effector function renders some subsets of T cells terminally differentiated in that those cells become fully committed to a specific differentiation program and will not revert to a pluripotent or a state of plasticity.

In addition to the development of effector T cells, some T cells differentiate into memory cells that are highly efficient at recall responses. There are several memory T cell subsets that have been described and these subsets can contribute to effective antitumor immunity. Central memory T cells have high proliferative capacity and are generally found in lymphoid tissues while effector memory T cells have limited proliferative capacity but swiftly gain effector function upon restimulation (Mueller et al., 2013). Within this subset are stem-cell memory cells identified in humans that express CD45RA, are multipotent, and have superior proliferative and self-renewing capacity (Gattinoni et al., 2009; Zehn et al., 2022). Tissue resident memory cells express tissue retention markers such as CD69 and CD103 and have a distinct transcriptional profile that is partly dictated by the tissue microenvironment (Schenkel and Masopust, 2014; Amsen et al., 2018; Pao et al., 2018; Szabo et al., 2019). The emergence and differentiation of memory T cells has traditionally been defined by a sufficient period of the absence of cognate antigen that allows for a resting period in memory formation. Memory cells are pluripotent with the ability to maintain their molecular signature and differentiate into effector cells when a specific antigen is subsequently recognized. Within the TME and in chronically infected tissues where antigen is constantly presented, new subsets of T cells with memory-like features that help to sustain the *in situ* immune response are being recognized.

## T cell entry and infiltration into the TME

Migration of effector T cells into tumors is orchestrated by interactions between chemokines, chemokine receptors, and adhesion molecules. Egress from the lymph nodes after T cell activation requires downregulation of lymph node homing molecules such as CD62L and CCR7 (Guarda et al., 2007; Groom et al., 2012) and the upregulation of sphingosine 1-phosphate receptor 1 (S1PR1) (Cyster and Schwab, 2012; Baeyens et al., 2015) and other chemokine receptors such as CXCR3 and CXCR6 (Groom and Luster, 2011; Karaki et al., 2021) and tissue-specific adhesion molecules (Mora and von Andrian, 2006; Ferguson and Engelhard, 2010). These changes in adhesion molecule expression permits the egress of activated T cells from lymphoid tissues and migration to peripheral sites. T regs may be more readily recruited into the TME because they express higher levels of various chemokine receptors such as CCR4, CCR5, and CCR8 compared to other T cell types (De Simone et al., 2016; Nishikawa and Koyama, 2021; Sasidharan Nair et al., 2021). Additionally, tumors that secrete CCL1 the ligand for CCR8 preferentially promote the recruitment of T regs into the TME (Barsheshet et al., 2017).

T cells extravasate into tissues at points of low shear stress in postcapillary venules (Choi et al., 2004) and endothelial cells lining vessels at peripheral tissue sites are key mediators of T cell migration and extravasation into the TME. Under ideal conditions, local inflammatory cues in the TME including TNF-α and IL-1 enhance the expression of chemokines and adhesion molecules in the microenvironment and on endothelial cells (Bellone and Calcinotto, 2013; Slaney et al., 2014; Peske et al., 2015). These changes allow binding of effector T cells to the vasculature and their extravasation into the TME. Interaction between selectins (carbohydrate binding molecules) such as E-selectin (CD62E) and P-selectin (CD62P) expressed on endothelial cells and their respective ligands, cutaneous lymphocyte antigen 1 (CLA-1) and P-selectin glycoprotein ligand 1 (PSGL-1), that are expressed on effector T cells results in slow rolling of the T cells on the vascular surface (McEver, 2015; Krummel et al., 2016). The slow rolling further allows chemokine receptors on the T cells to engage chemokines secreted by the endothelial cells. Engagement of the chemokine receptors then leads to changes in the conformational state of adhesion molecules from low affinity to high affinity states that permit stronger adhesive bonds between the T cells and the vasculature to support endothelial transmigration of the effector T cells into the TME (Bellone and Calcinotto, 2013).

### Modulation of adhesion molecules, chemokines, and chemokine receptors

**FIGURE 2:**
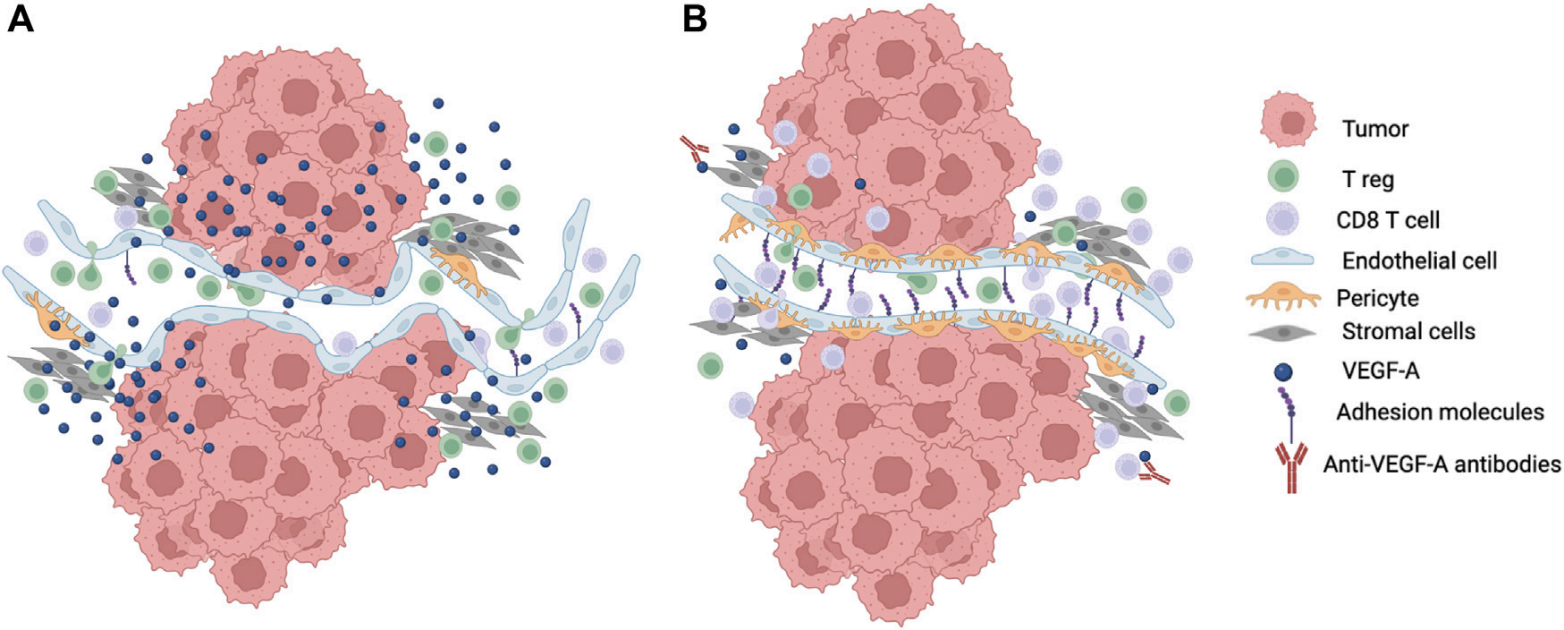
Modulation of T cell infiltration into the TME. **(A)** Growth and energetic demands of tumor cells creates a hypoxic TME that drive secretion of angiogenic factors such as VEGF-A into the microenvironment. This leads to neovascularization with new vessel formation that are immature, tortuous, easily compressed, and leaky with reduced pericytes and suboptimal surrounding basement membranes. The abnormal tumor vasculature contributes to the disruption of laminar flow and shear stress. Increased VEGF-A also leads to downregulation of adhesion molecules like ICAM-1 and VCAM-1 that are required for T cell adhesion and extravasation. Tumor cells may also preferentially secrete chemokines that attract T regs. These changes in the microenvironment lead to decreased effector T cell infiltration into the TME. **(B)** Reduction in the amount of VEGF-A in the TME such as through the blockade of VEGF-A using antibodies leads to normalization of the tumor vasculature with better perfusion and increased T cell adhesion to endothelial cells as well as less vascular compression and less mechanical stress that improve effector T cell infiltration into the TME. Created with Biorender.com.

Infiltration of T cells into the TME is dependent on the TME generating the appropriate inflammatory environment to active endothelial cells lining tumor associated vessels (Figure 2). However, even with the appropriate inflammatory signals in the TME, the tumor vasculature has been shown to be less responsive to inflammation leading to what has been described as endothelial anergy (Piali et al., 1995; Griffioen et al., 1996a; Weishaupt et al., 2007; Clark et al., 2008; Afanasiev et al., 2013).

Endothelial anergy has been partially attributed to angiogenic factors (Griffioen et al., 1996b; Dirkx et al., 2003) present in the TME that can be secreted by tumor, immune, and stromal cells (Hendry et al., 2016). Angiogenic factors such as vascular endothelial growth factor A (VEGF-A) and basic fibroblast growth factor (bFGF) are upregulated in many tumors partly because of hypoxic conditions in the TME (Keck et al., 1989; Leung et al., 1989). Intercellular adhesion molecule 1 (ICAM-1), ICAM-2 (Griffioen et al., 1996b), and vascular cell adhesion molecule 1 (VCAM-1) (Piali et al., 1995) expression on endothelial cells can be suppressed by VEGF-A and bFGF (Griffioen et al., 1998; Zhang and Issekutz, 2002). This results in the inhibition of T cell adhesion and extravasation. Consistent with this, investigators have shown that blocking antibodies against VEGF enhances infiltration of adoptively transferred T cells that translates into better tumor (Jain, 2005; Shrimali et al., 2010). Similarly, vascular E-selectin expression can be downregulated by angiogenic factors in the TME that results in decreased infiltration of T cells (Tromp et al., 2000; Maksan et al., 2004; Clark et al., 2008; Afanasiev et al., 2013).

The endothelin 1 and endothelin B receptor (ETBR) axis also interfere with ICAM-1 expression and T cell homing. Overexpression of ETBR on ovarian tumor endothelial cells suppresses ICAM-1 expression and induces nitric oxide synthase and nitric oxide (Tsukahara et al., 1994; Buckanovich et al., 2008). Increased nitric oxide in the endothelium also hinders ICAM-1 clustering thereby preventing T cell adhesion and extravasation (Buckanovich et al., 2008). Reactive nitrogen species in the TME promote chemokine nitration that also blocks T cell infiltration (Molon et al., 2011). Consequently, tumors with minimal to no TILs are associated with overexpression of ETBR and blocking ETBR leads to increased expression and clustering of ICAM-1 on endothelial cells and increased T cell infiltration into tumors (Buckanovich et al., 2008). In glioblastomas, vessels expressing ETBR have less T cell infiltrates in the surrounding area in comparison to areas with ETBR negative vasculature (Nakashima et al., 2016). The association between ETBR expression and TILs may be dependent on the type of tumor as other studies have failed to find an association between T cell infiltration and ETBR expression in oral squamous cell carcinoma (Tanaka et al., 2014).

### Structure and mechanics of tumor vasculature

The physical properties of vasculature in the TME also impacts T cells trafficking into tumors. New vessels are formed through a process called neovascularization in response to hypoxia and angiogenic factors secreted by tumor and stromal cells in the TME (Katayama et al., 2019). These vessels are generally immature, disorganized, tortuous, saccular, and leaky. Loose attachment or lack of pericytes, smooth muscle cells, thick or thin basement membrane around the vessels, increased fenestrations, and wide intercellular junctions between endothelial cells contribute to leakiness and abnormal tumor vasculature (Jain, 2005; Chung et al., 2010; Carmeliet and Jain, 2011; Farnsworth et al., 2014). Furthermore, tumor cells can mimic endothelial cells (Hendrix et al., 2003) creating pseudo-vessels that lack the properties of normal vasculature. The leakiness of the vasculature and compression of vessels by surrounding tumors also increase interstitial pressure in the microenvironment that disrupts perfusion thereby perpetuating a hypoxic environment (Heldin et al., 2004; Jain, 2013; Jain et al., 2014). The abnormal formation of vessels in the TME causes discontinuous laminar blood flow and points of low and no shear stress (Hendry et al., 2016). Additionally, endothelial cell polarity and the expression of adhesion molecules on endothelial cells is regulated in part by shear stress (Carmeliet and Jain, 2011). T cell rolling on endothelial cells requires a certain threshold of shear stress (Finger et al., 1996; Lawrence et al., 1997). Consequently, disruptions in shear stress negatively impact T cell rolling, adhesion, and extravasation.

## Immune cell niches in the TME that support T cell function

Chemokines, cytokines, tumor vasculature, and environmental cues direct T cell migration and localization within the TME. Heterogeneous distribution of these factors likely lead to discrete areas of immune cell aggregates within the TME. Inflammatory signals from factors such as TNF-α can lead to the remodeling of tumor vasculature and the upregulation of adhesion molecules, chemokines, and chemokine receptors that attract T cells and other immune cells into those perivascular areas. The cascade of events results in the formation of discrete clusters of immune and stromal cells that contribute to the localized immune response in the TME.

### Tertiary lymphoid structures

The presence of aggregates of immune cells in inflamed non-lymphoid tissues has been noted for many years. These aggregates are inducible, transient, and unencapsulated structures that range from simple clusters of lymphoid cells to well-organized immune structures with compartmentalized B and T cell areas intertwined in a stromal network and vasculature that closely resemble secondary lymphoid organs (SLOs) (Schumacher and Thommen, 2022). The well-organized aggregates are known as tertiary lymphoid structures (TLSs). While there is a range in the composition and organization of immune aggregates in inflamed non-lymphoid tissues, the current accepted definition of TLSs is the presence organized lymphocyte and myeloid cell aggregates in inflamed tissues with discernable B and T cell-like areas within these structures (Ruddle, 1999). TLSs have been identified in several diseases and conditions including infection, cancer, autoimmunity, and transplanted tissues (Hjelmstrom, 2001; Aloisi and Pujol-Borrell, 2006; Sato et al., 2009; Pitzalis et al., 2014; Hiraoka et al., 2015). They are thought to form through a process akin to that which governs the ontogeny of SLOs (Ruddle, 1999) and CXCL13-expressing effector CD8 T cells seem to play a major role in the induction of TLS formation (Workel et al., 2019; Rodriguez et al., 2021). TLSs are also functionally similar to SLOs with compartmentalized B cell-rich and T cell-rich areas and evidence of T and B cell activation and differentiation (Murakami et al., 1999; Moyron-Quiroz et al., 2006) and have been implicated in perpetuating autoimmune disease and allograft rejection as well as in helping to control infection and tumor growth (Hjelmstrom, 2001; Aloisi and Pujol-Borrell, 2006; Sato et al., 2009; Pitzalis et al., 2014; Hiraoka et al., 2015).

The functional significance of TLSs has largely been inferred from phenotypic evidence of T cell function in TLSs and correlative studies linking the presence of TLSs with increased T cell infiltration, tumor control, and prognosis in human patients. In melanoma tumor samples, T cell activation markers are increased in TLSs (Helmink et al., 2020). Naïve T cells have also been observed to proliferate within TLSs (McMahon et al., 2005; Lee et al., 2006). Germinal centers and upregulation of AICD, BCL6, and other markers of germinal center formation, somatic hypermutation, and class switching have also been identified in TLSs (Armengol et al., 2001; Sims et al., 2001; Nielsen and Nelson, 2012; Germain et al., 2014).

In addition to the correlative analyses, studies in mice lacking SLOs have provided data that suggest functional relevance of TLSs in the TME and in infection. In such models, induction of TLS formation has been associated with the presence of antigen-specific T cells and some level of disease control. For example, induction of TLS by lymphotoxin-α leads to T cell effector function and tumor control in mice that lack lymph nodes (Schrama et al., 2001; Schrama et al., 2008). Furthermore, mice lacking spleen, lymph nodes, and Peyer’s patches are capable of developing antigen-specific effector and memory T cells that are associated with inducible bronchus-associated lymphoid tissues, a type of TLS in the lung, when infected with influenza (Moyron-Quiroz et al., 2004; Moyron-Quiroz et al., 2006; Rangel-Moreno et al., 2007).

In support of the evidence suggesting the functional capacity and relevance of TLSs, several studies have shown an association between the presence of TLSs and overall survival and/or clinical responsiveness to different forms of cancer immunotherapy. While some studies have reported no effect or a negative impact of TLSs on immune response against cancer (Bento et al., 2015; Figenschau et al., 2015; Finkin et al., 2015; Joshi et al., 2015), there is strong and growing evidence to suggest that the presence of TLSs has a positive predictive value for patients. In several different tumor types, the presence of TLSs has been associated with responsiveness to immune checkpoint inhibition and better survival (Cabrita et al., 2020; Helmink et al., 2020; Petitprez et al., 2020). It is unclear whether immune checkpoint blockade drives *de novo* TLS formation; however, it can enhance the number of TLSs within tumors (Cottrell et al., 2018; van Dijk et al., 2020). On the other hand, vaccines and neoadjuvant chemotherapy can induce TLS formation in several tumor types (Lutz et al., 2014; Maldonado et al., 2014; Remark et al., 2016; Morcrette et al., 2019). In aggregate, the data strongly suggest that TLSs, immune niches within the TME, contribute to T cell function and the antitumor immune response.

## T cell exhaustion

As effector CD8 T cells engage tumor cells in the TME, they become dysfunctional over time and lose the ability to produce effector molecules such as cytokines and cytotoxic granules (Barber et al., 2006; Wherry, 2011). This state results from continued and persistent antigenic stimulation and is referred to as T cell exhaustion. There are two main subsets of exhausted T cells (terminally differentiated exhausted and stem-like, also known as progenitor exhausted cells) that have been described (Zehn et al., 2022). The terminally differentiated exhausted T cells express many inhibitory receptors collectively known as immune checkpoint molecules (Virgin et al., 2009). T cells in this state have limited to no capacity to kill tumor cells thereby hampering the quality of the immune response (Bengsch et al., 2016). Loss of effector function is progressive with the loss of the ability to produce IL-2, reduced proliferative capacity, and ineffective target cell killing occurring first (Wherry, 2011). This is followed by the decreased capacity to produce other cytokines such as TNF-α and IFNγ and then cell death.

The programmed cell death 1 (PD-1) molecule is the major mediator of T cell exhaustion. It is upregulated on T cells upon T cell activation and the expression of PD-1 on the cell surface can be detected very early in the T cell activation phase (Ahn et al., 2018). The induction of PD-1 expression is dependent on TCR-antigen/MHC interaction and inhibition of PD-1 signaling in the early T cell activation phase results in enhanced mTOR signaling and increased production of effector molecules (Ahn et al., 2018). Repeated antigen exposure and T cell stimulation leads to high levels and persistent PD-1 expression on activated T cells. T regs constitutively express high levels of PD-1 and PD-1 expression serves to promote proliferation of T regs (Francisco et al., 2009). PD-1 has two ligands, PD-L1 that is expressed on a wide variety of cells including many cancer cells, and PD-L2 that is expressed mainly on DCs (Keir et al., 2008). Engagement of PD-1 on effector T cells leads to diminished proliferation and cytokine production (Freeman et al., 2000; Carter et al., 2002; Brown et al., 2003). Blocking the interaction between PD-1 and its ligands results in the reinvigoration of T cells as evidenced by an expansion of effector cells and the recovery of cytokine production and cytolytic ability (Barber et al., 2006; Huang et al., 2017). PD-1 also regulates metabolism in T cells. Engagement of the PD-1 pathway plays a role in metabolic reprogramming of T cells by promoting fatty acid oxidation and limiting glycolysis, amino acid metabolism, and glutaminolysis (Parry et al., 2005; Patsoukis et al., 2015). Since effector T cells depend on glycolysis to maintain proliferation and cytokines, this change in metabolism compromises effector function.

As effector cells progress through the process of exhaustion, additional inhibitory molecules such as lymphocyte activation gene 3 (LAG-3), T cell immunoglobulin and mucin-domain containing-3 (TIM-3), and T cell immunoglobulin and ITIM domain (TIGIT) are upregulated. LAG-3 is a type I transmembrane protein that is structurally like CD4 (Triebel et al., 1990) and binds to MHC class II (Baixeras et al., 1992), galectin-3 (Kouo et al., 2015), α-synuclein (Mao et al., 2016), LSECtin (Xu et al., 2014a), and fibrinogen-like protein 1 (Wang et al., 2019). It is expressed on activated T cells and other immune cells and exists in transmembrane and soluble forms (Li et al., 2004). It colocalizes with CD3 in immune synapses to regulate TCR and calcium channel signaling to leads to inhibition of proliferation and cytokine production (Maçon-Lemaître and Triebel, 2005; Guy et al., 2022). TIM-3 is expressed on IFNγ-secreting T cells and engagement of TIM-3 with its ligands inhibits T cell function and induces apoptosis by regulating intracellular calcium flux (Zhu et al., 2005; Kang et al., 2015; Wolf et al., 2020). TIGIT is mainly expressed on T and NK cells and has two known ligands, CD155 and CD112 (Yu et al., 2009). TIGIT suppresses immune function by competing with costimulatory molecules CD266 and CD96 (Dougall et al., 2017). Interaction with CD155 on DCs results in IL-10 production that reduces effector function (Yu et al., 2009). It also negatively regulates T cell proliferation and inhibits IFNγ production (Lozano et al., 2012). Knockdown of TIGIT using short hairpin RNA restores T cell proliferation and cytokine production (Lozano et al., 2012). Many of these inhibitory molecules (ligands and receptors) are upregulated in the TME and are associated with poor prognosis (Zhang et al., 2017).

Given the negative effects of immune checkpoint molecules on effector T cell function and negative prognosis for patients with cancer, immune checkpoint molecules are being targeted for immunotherapy using different approaches that block the negative regulation these molecules exert on T cell function. Blockade of the inhibitory pathways in T cells leads to recovery of some, if not all, of the effector T cell function and has been associated with improved clinical response and outcome for patients with cancer (Brahmer et al., 2012; Topalian et al., 2012; Lipson et al., 2013). Inihibitors of mmune checkpoint molecules such as blocking antibodies against PD-1 and CTLA-4 are currently FDA approved and used routinely for the clinical management of cancer as monotherapeutic agents or in combination with other treatments (Twomey and Zhang, 2021) and many more are currently in clinical trials.

### Stem-like CD8 T cells

The second subset of exhausted CD8 T cells are known as stem-like CD8 T cells or progenitor exhausted cells and differentiate in chronic disease states such as chronic viral infections and cancer (He et al., 2016; Im et al., 2016; Utzschneider et al., 2016). These cells have pluripotent and memory-like features and are present in lymphoid and inflamed tissues as well as in the TME (Utzschneider et al., 2016; Im et al., 2020). They express PD-1, TCF-1, CD73, co-stimulatory molecules, low levels of CD127 and generally lack expression of other inhibitory molecules such as TIM-3 (Im et al., 2016). They have the capacity for self-renewal as well as the ability to differentiate into effector cells. As such, stem-like CD8 T cells sustain the terminally differentiated effector population in chronic infection and tumors and are essential for the maintenance of the effector response. Inhibition of the PD-1 pathway using blocking antibodies against PD-1 or its ligand, PD-L1, results in expansion of the stem-like CD8 T cells and differentiation into effector cells (Im et al., 2016; Hudson et al., 2019). This proliferative burst requires CD28 co-stimulation and stem-like CD8 T cells lacking CD28 fail to proliferate (Kamphorst et al., 2017). The expansion and generation of new effectors from stem-like CD8 T cells after PD-1 inhibition underscores the immunological and positive clinical responses seen in cancer patients treated with immune checkpoint inhibitors.

Of note, the T cell exhaustion molecular program, marked by upregulation of TOX and NR4A1, is maintained in stem-like CD8 T cells and is imprinted onto effector progeny derived from the stem-like cells (Utzschneider et al., 2013; Martinez et al., 2015; Pauken et al., 2016; Alfei et al., 2019; Khan et al., 2019; Scott et al., 2019; Seo et al., 2019; Yao et al., 2019). This programming cannot be overridden by using immune checkpoint inhibition to drive the differentiation of new effector progeny (Utzschneider et al., 2013; Pauken et al., 2016; Ghoneim et al., 2017; Philip et al., 2017). However, recent studies have shown that the combination of IL-2 and inhibition of the PD-1 pathway modifies the transcriptional program of effector progeny derived from stem-like CD8 T cells (Codarri Deak et al., 2022; Hashimoto et al., 2022). This effect is mediated by the high affinity IL-2 receptor, CD25. Stem-like CD8 T cells transferred into a new host that receive IL-2 stimulation in the presence of PD-1 blockade produce progeny with a molecular profile distinct from the exhaustion pathway with downregulation of Tox, Nr4a1, other genes in the exhaustion pathway, and several inhibitory receptors. The molecular profile of effector cells that differentiate in this context resemble highly functional effector cells that are generated from acute viral infections. Thus, the cytokine milieu during reactivation and expansion of stem-like CD8 T cells in lymphoid tissues and the TME can significantly impact the quality of the immune response.

## Metabolism and nutrients in the TME

Several metabolic pathways regulate T cell differentiation and effector function. Resting T cells use oxidative phosphorylation to fuel ATP production. Once activated, downstream signaling pathway feeding through the P13K/AKT/mTOR pathway promotes Myc-dependent metabolic reprogramming to glycolysis (Araki et al., 2009; MacIver et al., 2011; Wang et al., 2011; Verbist et al., 2016). This switch in metabolism is required for the high energy demands for T cell expansion and the production of effector molecules such as IL-2, IFNγ, and granzyme B (Cham et al., 2008; Chang et al., 2015). Short-lived effector T cells such as terminally differentiated cells also depend on glycolysis. In contrast, T regs and memory T cells depend on oxidative phosphorylation and fatty acid metabolism. In T regs, fatty acid oxidation and oxidative phosphorylation promote immunosuppressive activity and these metabolic pathways are driven by FOXP3 (Michalek et al., 2011; Angelin et al., 2017). In fact, the expression of the enzymes required for glycolysis can be suppressed by FOXP3 (Angelin et al., 2017). T regs also upregulate molecules involved in fatty acid transport such as CD36 and sterol-regulatory-element binding protein that supports the use of lipids in the TME for proliferation and immunosuppressive activity (Wang et al., 2020; Lim et al., 2021). Consistent with this, an accumulation of T regs has been noted in tumors that produce fatty acids such as gastric cancer cells with RHOA Y42 mutation (Kumagai et al., 2020). In TMEs with low levels of fatty acids, T regs upregulate fatty acid binding proteins to scavenge for nutrients to support the immunosuppressive function (Field et al., 2020).

High metabolic activity of tumor cells that maintains growth depletes nutrients in the TME leaving limited supply of glucose for activated T cells to maintain proliferation and adequate effector function (Kishton et al., 2017). Additionally, the abnormal vasculature in the TME results in heterogenous distribution of nutrients that further compromises effector T cell metabolism. Limited nutrients in the TME, in addition to chronic stimulation, result in changes in the metabolic profile of effector T cells that lead to reduced glucose uptake, increased expression of reactive oxygen species, loss of mitochondrial mass, and reduced effector function in T cells (Scharping et al., 2016; Siska et al., 2017). To adapt to the limited nutrients in the TME, activated T cells can incorporate other biomolecules like glutamine and amino acids into mitochondrial respiration to support proliferation and effector function (Michalek et al., 2011; Sinclair et al., 2013; Johnson et al., 2018). T cells can breakdown glutamine into a-ketoglutarate and other intermediates that feed into the TCA cycle (Johnson et al., 2016). However, some cancer cells do not express the enzymes required for glutamine synthesis (Cluntun et al., 2017) and therefore also compete with T cells for exogenous glutamine. Activated T cells can also utilize endogenous and exogenous lipids for β-oxidation to maintain proliferation and function (MacIver et al., 2013; Howie et al., 2017). However, the use of other metabolic pathways may not be as effective as glycolysis in promoting effector function in the TME as suggested by studies showing insufficient production of cytokines when T cells are forced to use other pathways such as oxidative phosphorylation (Chang et al., 2013). Other molecules such as methionine and arginine can also be depleted by cancer cells and myeloid-derived suppressor cells in the TME that negatively impact T cell proliferation effector function (Rodriguez et al., 2003; Bian et al., 2020; Grzywa et al., 2020).

Metabolic byproducts produced in the TME can also impact T cell proliferation and effector function. The most widely recognized metabolite in the TME to negatively impact T cells is lactate or lactic acid that is transported from cancer cells as a byproduct of glucose metabolism (Halestrap and Wilson, 2012). Lactate hinders proliferation and cytokine production by depleting intracellular levels of nicotinamide adenine dinucleotide (NAD^+^) (Fischer et al., 2007). T regs are generally unaffected by the effects of lactic acid on intracellular NAD^+^ because mitochondrial metabolism in T regs generates NAD^+^ (Angelin et al., 2017). Additionally, PD-1 expression on T regs can be upregulated in response to lactic acid in the TME and this enhances the immunosuppressive activity of T regs (Kumagai et al., 2022). Increased levels of lactate in the TME also lowers the pH within tumors, which poses further restrictions on cytokine production and the cytolytic activity of T cells (Fischer et al., 2007; Erra Díaz et al., 2018). Consequently, neutralization of the acidic pH in murine tumors with sodium bicarbonate leads to increased sensitivity of tumors to immune checkpoint blockade therapies and reduction of tumor volume (Pilon-Thomas et al., 2016).

## Hypoxia

Rapidly growing cancer cells have high oxygen consumption requirements that create hypoxic environments within tumors. Tortuous and abnormal tumor vasculature can also result in poor perfusion of the TME leading to low oxygen levels, especially in the central tumor bed, that can impact T cell function. Hypoxia triggers the activation of the hypoxia-inducible factor-1 (HIF-1) pathway and upregulation of HIF-1 proteins. Activation of this pathway influences the fate of differentiating CD4 T cells by promoting the polarization of the cells into the proinflammatory T_H_1 and T_H_17 subsets of helper cells (Dang et al., 2011). Additionally, HIF-1 can directly promote gene transcription of FOXP3 in a process mediated by TGF-β (Ren et al., 2016). In ovarian and hepatocellular tumors, hypoxia can drive the increased secretion of CCL28 that leads to increased recruitment of CCR10-expressing T regs into the TME (Facciabene et al., 2011; Ren et al., 2016). A similar effect of hypoxia on T reg migration involving CXCL12 and CXCR4 has also been reported in breast cancer (Yan et al., 2011). Within the TME, sustained exposure to hypoxia has been shown to promote the progression of effector CD8 T cells into terminally differentiated cells with an exhausted phenotype. In TMEs with severe hypoxia such as tumors with increased oxidative metabolism, the exhausted T cells are unresponsive to immune checkpoint inhibitors (Najjar et al., 2019). In hypoxic conditions, MHC class II and co-stimulatory molecules such as CD86 and CD40 expression on DCs have been found to be downregulated (Correale et al., 2012). Additionally, hypoxia negatively impacts chemotaxis to CCR5 and CCR4 ligands by monocyte-derived DCs (Labiano et al., 2015). As discussed earlier, increased levels of VEGF-A, which can be induced by hypoxia, in the TME inhibits DC maturation that results in ineffective T cell stimulation (Noman et al., 2015). Overall, hypoxia adds to the hostile microenvironment that limits effective T cell activation, effector function, and survival.

## T cell survival in the TME

Maintenance of a robust antitumor immune T cell response is essential to control tumor growth. This can be accomplished by continued replenishment of effector T cells in the TME through the activation and migration of new effector cells from lymphoid tissues, the activation and differentiation of naïve T cells within the TME, and/or supporting T cell survival in the TME. Effector T cells upregulate pro-apoptotic receptors such as FAS and TNFRs upon activation, which leaves them vulnerable to apoptosis (Singer and Abbas, 1994; Ettinger et al., 1995; Zheng et al., 1995). Progressive differentiation of T cells also leads to the telomere shortening (Weng et al., 1995) that decreases lifespan and promotes senescence. However, the main factors that influence T cell longevity are cytokines and metabolism (Chang and Pearce, 2016; Kishton et al., 2017; Pan et al., 2017; Lin et al., 2020).

Several cytokines promote T cell survival. The IL-2 family of cytokines that includes IL-2, IL-4, IL-7, and IL-15 has a major influence on T cell lifespan. They are expressed by a variety of immune cells as well as epithelial and endothelial cells (Benczik and Gaffen, 2004) and they can regulate T cell survival independent of their capacity to induce proliferation and in part, through the upregulation of anti-apoptotic genes such as BCL2 (Marrack et al., 1998; Vella et al., 1998; Zhang et al., 1998). However, T cells may respond to these environmental cues differently depending on their differentiation state. T cell sensitivity to cytokines can also vary between resting (naïve and memory) T cells and activated/effector T cells. While IL-6 supports the survival of resting T cells, it has little effect on the longevity of activated T cells (Teague et al., 1997). Activated T cells rely on IL-2 and IL-4 signaling for survival and withdrawal of these cytokines lead to T cell death (Marrack et al., 1998; Borthwick et al., 2003). Sequestration of IL-2 by T regs in the TME (Ku et al., 2000; Carmenate et al., 2018) limit the availability of the cytokine for other T cell subsets and thereby impede their survival.

Interestingly transmigration of activated T cells across endothelial cells can support T cell survival through the interaction between ICAM-1 and LFA-1 in the absence of IL-2 (Borthwick et al., 2003). Effector T cells are also more sensitive to survival cues from Type I interferons secreted in inflammatory conditions than resting cells (Marrack et al., 1998). In addition to supporting the survival of memory T cells, IL-15 can also rescue activated T cells from death (Vella et al., 1998; Zhang et al., 1998; Di Pilato et al., 2021). A subset of DCs known as DC3 or LAMP3+ DCs support T cell survival in the TME by secreting CXCL16 to attract CXCR6-expressing T cells into perivascular areas in the TME where CCR7+ DC3 are clustered (Di Pilato et al., 2021). In these clusters, the DCs provide IL-15 stimulation to the T cells to prevent activation-induced cell death. Notably, DC3s do not express XCR1, the receptor for XCL1 that is secreted by stem-like CD8 T cells (Im et al., 2016) and it is not known if other subsets of DCs such as conventional DC1s that express XCR1 are also capable of trans-presenting IL-15 to different subsets of T cells.

In addition to cytokines, specific metabolic and catabolic pathways can regulate T cell survival. Lipid uptake and fatty acid metabolism supports T cell survival. Resident memory T cells deficient in fatty acid-binding proteins 4 and 5 reduces lipid uptake and impairs survival (Pan et al., 2017). Blockade of mitochondrial fatty acid β-oxidation also reduces the lifespan of resident memory T cells. However, the survival of central memory T cells is not impacted in the absence of FABP4 and FABP5 suggesting that different subsets of T cells rely on distinct cues for survival. In contrast to memory T cells, chronic mTOR signaling in effector T cells enforces glycolysis and amino acid metabolism that render the cells vulnerable to cell death (Araki et al., 2009; MacIver et al., 2011; Pollizzi et al., 2016; Verbist et al., 2016). Consequently, inhibition of mTOR signaling improves T cell persistence (Araki et al., 2009). When exposed to high levels of L-arginine, metabolism in activated T cells shifts from glycolysis to oxidative phosphorylation that drives better survival (Geiger et al., 2016). Autophagy has also been shown to impact T cell longevity. T cell survival and memory formation are defective in T cells that lack autophagy related 4 and 5 genes (Xu et al., 2014b). These data show that the mechanisms that support T cell survival in the microenvironment are complex and distinct for different subsets of T cells in the TME. As investigators elucidate critical factors that regulate T cells in the TME, targeted therapeutic approaches that promote T cell survival may further enhance clinical efficacy of cancer immunotherapy.

## Therapeutic applications and opportunities

Therapeutic strategies for cancer immunotherapy span all stages of the adaptive immune response and include vaccine development, adjuvant, immune modulators, and adoptive T cell therapy. Cancer vaccines involve regimens consisting of tumor antigens alone or in combination with adjuvants. Tumor antigens can be delivered as vaccines in a variety of formulations. These include whole tumor lysates, peptides, in DNA and mRNA-based constructs, and peptides loaded onto DCs (Saxena et al., 2021). There are also several therapeutic strategies targeting DC activation and maturation to induce robust antitumor T cell responses. Strategies and adjuvants that stimulate toll-like receptor pathways such as polyICLC and CpG that induced DC maturation and activation are being investigated (Chiang et al., 2013; Woo et al., 2014; Barber, 2015; Liau et al., 2018; Ramanjulu et al., 2018).

Previous efforts for cancer vaccines have been focused on shared tumor antigens derived from overexpressed self-antigens, differentiation proteins, and cancer testis antigens and these efforts have resulted in limited efficacy for a subset of cancer types (Comiskey et al., 2018; Pavlick et al., 2020). Although the intent of using shared tumor antigen is to develop vaccines that can be broadly used against several tumor types and for different patients, the tumor antigen profiles that can elicit strong and protective immune responses can be distinct between patients and tumor types. As such, there is now a focus on strategies utilizing individualized neoantigens as therapeutic vaccines (Carreno et al., 2015; Ott et al., 2017; Sahin et al., 2017; Keskin et al., 2019) as well as a combination of shared antigens and personalized neoantigens (Hilf et al., 2019; Saxena et al., 2021). High tumor mutational burden correlates with the presences of neoantigens and clinical response to immune checkpoint inhibitors (ICIs) (Maleki Vareki, 2018; Miao et al., 2018). Therapeutic approaches combining neoantigen vaccines with ICIs are being pursued (Ott et al., 2020; Saxena et al., 2021). Prophylactic vaccines targeting pathogen-mediated/driven tumors such as human papillomavirus-associated cervical cancer and oropharyngeal squamous cell carcinoma have also shown significant efficacy in reducing the risk of cancer in patients (Melief et al., 2015; Tomaić, 2016; van der Burg et al., 2016).

Immune modulators are a class of cancer immunotherapeutic agents that are used to induce and support antitumor responses. Immunomodulators include adjuvants, cytokines, chemokines, costimulatory agonists, and ICIs. IL-2 and IFNα were the first immunomodulators used to treat melanoma and renal cell carcinoma (Waldmann, 2018), and molecules that target the IL-2 and IFNAR pathways are FDA-approved for some cancer types. Other cytokines are being investigated to support T cell function and survival include IL-12, IL-15, and IL-21 (Floros and Tarhini, 2015). Co-stimulatory agonists for CD40, CD137 (4-1BB), OX-40 and others are currently in clinical trials to treat different cancer types (Mayes et al., 2018). Several ICIs against PD-1, PD-L1, CTLA-4, and LAG3 are FDA-approved therapies for several different types of cancers including melanoma, lung, gastrointestinal, breast, and renal cell cancer (Vaddepally et al., 2020). Other immunosuppressive factors including indoleamine 2, 3-dioxygenase (IDO) (Zhai et al., 2018; Kjeldsen et al., 2021) and the adenosine pathway (Leone and Emens, 2018; Fong et al., 2020) are also being targeted for cancer therapy.

Adoptive T cell therapy involves the transfer of expanded tumor infiltrating lymphocytes or engineered T cells to reduce tumor burden. The isolation, *in vitro* expansion, and reinfusion of tumor infiltrating lymphocytes into patients as personalized therapy have resulted in good durable clinical responses (Hinrichs and Rosenberg, 2014) and adoptive T cell therapy is a viable approach to treat some types of cancers. T cells can also be engineered to express distinct tumor-specific T cell receptors that can then be infused into patients. Engineered T cells with a specific T cell receptor against the cancer testis antigen New York esophageal squamous cell carcinoma 1 (NY-ESO-1) have been used to treat solid tumors (D'Angelo et al., 2018; Robbins et al., 2011). Chimeric antigen receptor (CAR) T cells that are engineered to recognize tumors and specific proteins (June et al., 2018) have demonstrated strong clinical efficacy for hematologic cancers (Huang and Huang, 2022) and can be modified to target other tumor types.

## Conclusion

T cell function in the TME is complex and influenced by a myriad of factors at every step of the life cycle of a T cell. These factors include direct cellular interactions with tumor, stromal, and other immune cells, structural and architectural features of the TME, soluble factors such as chemokines and cytokines, nutrients and metabolites, and metabolism in the TME. The resulting functional phenotype of effector T cells in the microenvironment is likely dictated by the sum and balance of opposing signals and cues in the TME. This poses challenges in developing robust therapeutic strategies with strong clinical efficacy. Despite these challenges, cancer immunotherapy has revolutionized clinical care and management of patients with cancer. There is an active and robust research community studying combinatorial approaches to bolster cancer treatment and many of the factors and cellular pathways that mediate T cell function provide new opportunities for innovation in cancer immunotherapy and in synergizing with other treatment modalities such as chemotherapy and radiation therapy.
